# Conformation and Catalytic Properties Studies of *Candida rugosa* Lip7 via Enantioselective Esterification of Ibuprofen in Organic Solvents and Ionic Liquids

**DOI:** 10.1155/2013/364730

**Published:** 2013-12-05

**Authors:** Xiang Li, Shuangshuang Huang, Li Xu, Yunjun Yan

**Affiliations:** Key Laboratory of Molecular Biophysics of the Ministry of Education, College of Life Science and Technology, Huazhong University of Science and Technology, Wuhan 430074, China

## Abstract

Enantioselective esterification of ibuprofen was conducted to evaluate the enzyme activity and ees of lipase from *Candida rugosa* (CRL7) in ten conventional organic solvents and three ionic liquids. Different alcohols were tested for selecting the most suitable acyl acceptor due to the fact that the structure of alcohols (branch and length of carbon chains; location of –OH functional group) could affect the enzyme activity and ees. The results of alcohol and solvent selection revealed that 1-isooctanol and isooctane were the best substrate and reaction medium, respectively, because of the highest enzyme activity and ees. Compared with the control, conformational studies via FT-IR indicate that the variations of CRL7's secondary structure elements are probably responsible for the differences of enzyme activity and ees in the organic solvents and ionic liquids. Moreover, the effects of reaction parameters, such as molar ratio, water content, temperature, and reaction time, in the selected reaction medium, were also examined.

## 1. Introduction

Recently, the enzyme-catalyzed biotransformation in micro-/nonaqueous solvents has become the exciting field of enzymology [1]. Their usage is especially suitable for the substrates that are unstable or poorly soluble in water [2]. Furthermore, at low moisture content, many water-dependent side reactions can be effectively suppressed [3]. However, the activity and stability of enzymes do not always match the requirement of reactions in micro-/nonaqueous medium. Klibanov reported that the activity and stability of lipases in reaction medium are mainly determined by their native structure. Their activity variations in non-aqueous media could mainly be ascribed to the corresponding change of enzyme conformation [4]. Therefore, it is very important to elucidate the correlation between lipase activity and its conformation variation in the reaction media, which would be better for the understanding of enzymatic biotransformation in non-aqueous medium.

In this work, CRL7 was chosen for evaluating the correlation between its structure and catalytic properties, for CRL7 has been extensively demonstrated to be effective for biotransformation reactions in aqueous and non-aqueous phases owing to its high activity and broad specificity. The yeast *C. rugosa* has a family of functional genes encoding several isoenzymes with closely related sequences named Lip1 to Lip7 [5]. Moreover, a novel lipase gene, *lipJ08*, was cloned from *C. rugosa* ATCC14830 in our laboratory [6]. Although CRL7 has been reported to be applied in many fields, such as enrichment of polyunsaturated fatty acids [7], biocatalytic synthesis of phytosterol esters [8], biodiesel synthesis [9], and even resolution of enantiomers [10], the relationship between its enzyme activity, especially enantioselectivity and conformation (secondary structure) variation, in the resolution reaction, has rarely been addressed. In particular, the comparison of enzyme activity/ees in conventional organic solvents and ionic liquids as well as its conformation variation in these media had seldom been studied.

The enantioselective esterification of ibuprofen with short chain alcohol was chosen in this study, as ibuprofen (2-(4-isobutylphenyl) propionic acid) is representative of the 2-aryl propionic acid (2-APA) derivative family. The 2-APA class of nonsteroidal anti-inflammatory drugs (NSAIDs) is one of the most commercially successful and important classes of analgesic anti-inflammatory drugs in the world [11]. They have an asymmetric carbon in the second position. The anti-inflammatory and analgesic effects of the 2-APA are attributed almost exclusively to the *S*-enantiomer by inhibiting cyclooxygenase system [12]. It has been reported that (*S*)-ibuprofen is 160-fold more active than its antipode in the synthesis of prostaglandin “in vitro” [13].

Therefore, the main purposes of this work are (1) to investigate the properties of CRL7 with different short chain alcohols and thus select the best acyl donor; (2) to examine the effect of various organic solvents and ionic liquids on the lipase structure and enzyme activity; and (3) to further explore the effects of reaction parameters, such as molar ratio, water content, temperature, and reaction time.

## 2. Materials and Methods

### 2.1. Materials

Racemic and optically pure ibuprofen was purchased from the National Institute for Food and Drug Control (China). CRL7 was bought from Sigma-Aldrich Co., Ltd (St. Louis, MO, USA). All organic solvents used were obtained commercially from Sinopharm Chemical Reagent Co., Ltd, Shanghai, China. Other reagents were of analytical grade. High-performance liquid chromatography (HPLC) grade organic solvents were got from TEDIA (USA).

### 2.2. Enzyme Activity Assay

According to the method described by Chen et al. [14], one unit (U) of enzyme activity was defined as the amount of the enzyme which produces 1 *μ*mol ibuprofen ester (isooctyl ester or other esters of short chain alcohols) per hour under the assay conditions. The reactions were performed in a 50 mL stoppered flask at 50°C and 200 rpm. The assay conditions were used except when otherwise stated in the text. Protein content determination of the lipase was determined by the method of Bradford [15].

### 2.3. Reaction Procedure

Before usage, both organic solvent and short chain alcohols were dried over 4 Å molecular sieves. 0.01 mmol ibuprofen and 0.1 mmol short chain alcohol were added 5 mL organic solvent. The reaction mixture reacted in a shaking bath for several hours at 37°C and 200 rpm. After addition of 100 mg CRL7, the mixture was incubated on a shaker at the same conditions. When the reaction ended, the lipase was then removed by filtration. Samples were taken for analysis by HPLC.

### 2.4. Analysis and Calculation

The samples were tested by HPLC (Model 2300-525 SSI. Co., Ltd., USA) using a chiral column (Chiralcel OD-H, 4.6 mm × 250 mm, Daicel, Japan) with hexane/2-propanol/trifluoroacetic acid (90 : 10 : 0.1, v/v; 1.0 mL/min) as mobile phase and detected at a wavelength of 254 nm (Model 525 UV Detector SSI. Co., Ltd., USA). The retention times of (*R*)- and (*S*)-ibuprofen in the column were 7.28 and 8.23 min, respectively.

Enantioselectivity was expressed as *E* value and was calculated by (1), ees (the enantiomeric excess of the substrate) was calculated by (2), and *C* was calculated by (3). Consider the following:

(1) $$\begin{array}{c} E=\frac{\ln\left[\left(1-C\right)\left(1-\text{ees}\right)\right]}{\ln\left[\left(1-C\right)\left(1+\text{ees}\right)\right]}, \end{array}$$

(2) $$\begin{array}{c} \text{ees}=\frac{S-R}{S+R}, \end{array}$$

(3) $$\begin{array}{c} C=\frac{S_{0}+R_{0}-\left(S+R\right)}{S_{0}+R_{0}}, \end{array}$$

where *C* represents the conversion ratio of the substrate, ees represents the enantiomeric excess of the substrate, *S*_0_ and *R*_0_, respectively, represent the concentrations of the *S*- and *R*-enantiomers of ibuprofen before reaction, and *S* and *R* represent the concentrations of the *S*- and *R*-enantiomers of ibuprofen after reaction.

### 2.5. FT-IR Spectroscopy

The CRL7 after being treated with organic solvent and ionic liquids was mixed with KBr and pressed into pellets, respectively. Then, the above samples were used in the FT-IR measurements. FT-IR measurements were conducted in the region of 400–4000 cm^−1^. The measurement conditions were 25°C, 20 kHz scan speed, 4 cm^−1^ spectral resolution, and 128 scan coadditions. The model of equipment was Vertex 70 FT-IR spectrometer (Bruker Optik GMBH, Germany) with the nitrogen-cooled, mercury-cadmium-tellurium (MCT) detector. The infrared spectrum of KBr was subtracted from the infrared spectrum during each measurement. The absorbance spectra at amide I band are between 1700 and 1600 cm^−1^ [16, 17]. The predominant absorbance spectra in amide I band were *α*-helix: 1650–1658 cm^−1^, *β*-sheet: 1620–1640 cm^−1^, *β*-turn: 1670–1695 cm^−1^, and random coli: 1 640–1 650 cm^−1^, respectively [16]. The secondary structure element content was estimated according to the method described by Yang et al. [18].

## 3. Results and Discussion

### 3.1. Alcohol Selection for the Enantioselective Esterification of Racemic Ibuprofen

To select the best acyl donor, different alcohols were employed to study the effects of alcohols on the enantioselective esterification of racemic ibuprofen. The results were shown in Figure 2.

As can be seen, almost all alcohols (except for *tert*-alcohols and 1, 2-ethanediol) brought about the esterification of ibuprofen. Compared with other primary alcohols, the enzyme activity and ees of straight chain C1–C3 alcohols were markedly lower than those alcohols with middle chain length (C4–C10 alcohols), which indicated that short chain alcohols had negative effect on lipase [19]. The enzyme activity and ees of straight chain C9-C10 alcohols were higher than other C4–C8 alcohols, and C4-C5 alcohols were higher than C6–C8 alcohols. These results indicated that the enzyme activity and ees were profoundly affected by the carbon chain length of alcohols, but the correlation between them was not linear. Among straight chain alcohols, the highest enzyme activity occurred in 1-decanol and its corresponding ees was 0.84 ± 0.03. However, the enzyme activity and ees of branch chain monohydric alcohols (isobutanol, isoamylol, and isooctanol) were more than those of their corresponding straight chain monohydric alcohols (1-butanol, 1-pentanol, and 1-octanol). Therefore, the enzyme activity and ees are not only dependent on the –OH functional group of alcohols, but also on its location and the structure of the carbon chains. Nevertheless, the esterification of ibuprofen from polyols and tertiary alcohols could not be detected, indicating that the substrate could not react with these alcohols, as the structure of carbon chain of polyols and tertiary alcohols caused more steric hindrance [20]. From Table 1, isooctanol was recommended for the suitable substrate because of its highest enzyme activity and ees.

### 3.2. Effects of Organic Solvents and Ionic Liquid on the Enzyme Activity and ees of CRL7 in the Resolution of Racemic Ibuprofen

Laane et al. reported that the log⁡⁡*P* has the fundamental effect of polarity-hydrophobicity of organic solvents on enzyme-catalyzed reaction [21]. The enzyme activity, stability, and even enantioselectivity in organic solvents are often correlated with the solvent hydrophobicity [20]. The higher activities were found when the log⁡⁡*P* was above 2 [22]. As shown in Table 2, the enzyme activity and ees could not be detected in organic solvents with log⁡⁡*P* < 2 (acetonitrile), which indicates that ibuprofen could not react with alcohol in this solvent. When log⁡⁡*P* of solvent was beyond 2, the enzyme activity and ees in alkanes (from cyclohexane to *n*-undecane, log⁡⁡*P* > 3) were much higher than those in xylene (log⁡⁡*P* = 2.5), which shows that solvents with higher hydrophobicity are more suitable for CRL7. However, the increase of log⁡⁡*P* of solvents did not have the same tendency for the enzyme activity and ees. Among alkanes, the highest enzyme activity occurred in isooctane, and its corresponding ees was 0.92 ± 0.01, which was also the highest value. Three different types of ionic liquids were also chosen as solvents. Compared with organic solvents, enzyme activity and ees in BmimPF6 and BmimTF2N were similar to those in *n*-undecane and *n*-nonane. Among ionic liquids, their enzyme activity and ees were not the same, which indicates that the cations and anions have different effects. This could also be proved by the results from Table 2: BmimPF6 and BmimTF2N had the same cation (Bmim) and BmimPF6 and EmimPF6 had the same anion (PF6), but their enzyme activity and ees were not the same. This phenomenon coincides with the result reported by Pan et al. who pointed out that the probable reason was ascribed to the viscosity and hydrophilicity of ionic liquids [23].

### 3.3. Secondary Structure Analysis of CRL7 by FT-IR Spectroscopy

Conformational structure change of the CRL7 treated with the organic solvents and ionic liquids is probably the reason for the variation of enzyme activity and ees [24]. To verify this hypothesis, CRL7 was incubated in organic solvents and ionic liquids with the same conditions as described above in Section 2.2. The organic solvents and ionic liquids were then removed under reduced pressure by a vacuum pump and the residual enzyme was dried according to the method described by Pan et al. [23]. Then, FT-IR experiments were conducted to analyze the secondary structure variation with the conditions described in Section 2.5, and the variations of secondary structure elements were shown in Table 3.

As can be seen in Table 3, the secondary structure element content of CRL7 without treatment of organic solvent and ionic liquids was *α*-helix: 43.46%, *β*-sheet: 26.91%, *β*-turn: 11.83%, and random coli: 17.79%, respectively. After being treated with organic solvents, the corresponding contents were *α*-helix: 21.03–40.88%, *β*-sheet: 22.22–46.43%, *β*-turn: 11.93–21.57%, and random coli: 17.94–25.83%. Compared with the control, on the whole, CRL7 exhibited a decrease in *α*-helix and an increase in *β*-sheet, *β*-turn, and random coil (except for xylene and acetonitrile whose *β*-sheet decreased). The decrease tendency in *α*-helix coincides with the conclusion of Pan et al. [23], who had reported that the enzyme activity increased with the decrease of *α*-helix content, which was ascribed to the influence of *α*-helix on the “open” tendency of active site of the lipase. The open tendency of active site would make it easier for the substrate to access it. Moreover, *α*-helix content treated by isooctane was the highest among organic solvents (except for acetonitrile), whose ees and enzyme activity were also the highest (see Table 1). As for *β*-Sheet, compared with the control, its content increased in short chain alkanes with relatively higher log⁡⁡*P* (from *n*-undecane to cyclohexane) and decreased in xylene (log⁡⁡*P* = 2.5) acetonitrile (log⁡⁡*P* = −0.33). Moreover, as shown in Table 1, the enzyme activity and ees in the short chain alkanes were much higher than those in xylene and acetonitrile (whose *β*-sheet decreased in Table 3). From the relationship between CRL7 activity and the corresponding *β*-sheet content, it was maybe speculated that the increase in *β*-sheet content of secondary structure in the solvents was responsible for their activity enhancement.

Compared with the control, random coil content decreased in all solvents, which had the opposite tendency of *α*-helix. The reason for the increased tendency was attributed to a certain amount of *α*-helix being converted into random coils. Zheng et al. reported that the conformational transition of lipase could lead to the decrease in *α*-helix and the increase in random coil [25].

The change of secondary structure element contents in ionic liquids was similar to the tendency of those in organic solvents: decrease in *α*-helix and increase in *β*-turn and random coil. Moreover, the variation ranges of *α*-helix and *β*-turn contents were not exceeding those in the short chain alkanes, which was in accordance with the variation trends of enzyme activity and ees. Gu and Li had pointed out that the activity variation of lipase in ionic liquids was probably related to its conformation change caused by different properties of ionic liquids, such as polarity, hydrophobicity, hydrogen bonding, basicity, and viscosity [26].

### 3.4. Effect of Reaction Parameters

#### 3.4.1. Effect of Molar Ratio

**Figure 1:**
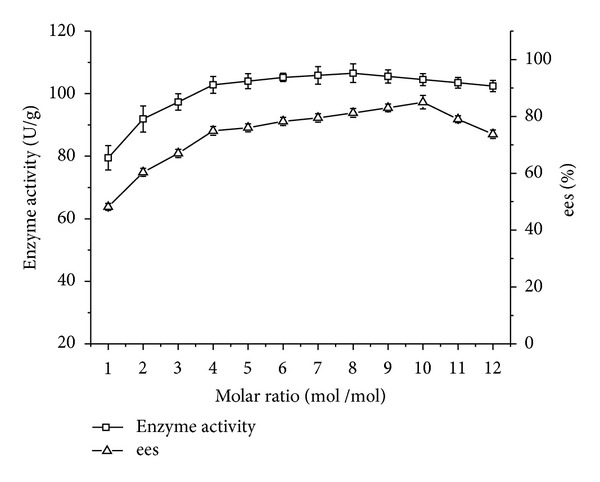
Effect of substrate molar ratio on enzyme activity/ees of CRL7. Reaction condition: 0.1 g CRL7 was added to 5 mL isooctane containing 0.1 mmol ibuprofen, 1–12 mmol isooctanol. The reactions were performed at 50°C, 200 rpm for 24 h. The data were measured in triplicate and vertical bars represent standard deviation.

According to the results from Table 1, isooctanol was chosen as acyl acceptor. 1 mol of isooctanol is required to react with 1 mol of ibuprofen. In practice, an excess amount of alcohol can drive the reversible reaction to the right side so as to produce more esters. As shown in Figure 1, both enzyme activity and ees had the same increasing tendency with the growth of molar ratio. For enzyme activity, its highest value was obtained at molar ratio of 8 : 1. For ees, the highest value occurred at molar ratio of 10 : 1. Beyond the highest value, both enzyme activity and ees showed a decrease tendency with further increase of molar ratio.

#### 3.4.2. Effect of Water Content

**Figure 2:**
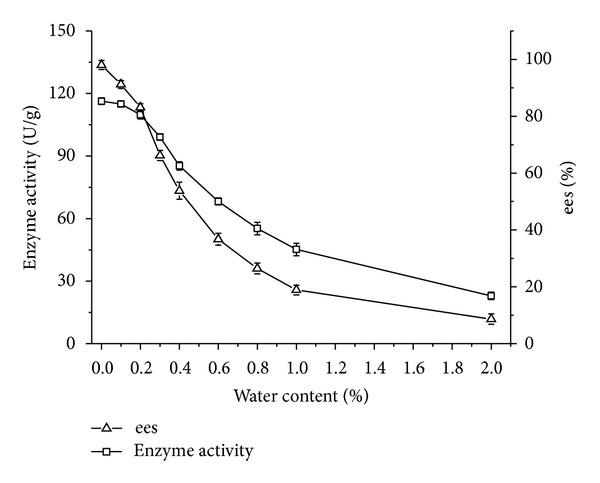
Effect of water content on enzyme activity/ees of CRL7. Reaction condition: 0.1 g CRL7 was added to 5 mL isooctane containing 0.1 mmol ibuprofen, 1 mmol isooctanol. The reactions were performed at 50°C, 200 rpm for 24 h. The data were measured in triplicate and vertical bars represent standard deviation.

Water plays a critical role in the structure and function of enzymes because of its influence on enzymes' active conformation. As can be seen in Figure 2, enzyme activity and ees show a decreasing tendency. Herbst et al. reported that protein destruction might take place when clusters of water on protein surface agglomerated into large clusters. These clusters caused structural changes by promoting the formation of enzyme agglomeration up to denaturation [22]. Therefore, if water layer is sufficiently large, the transfer of acyl group to the active site will be prevented, which leads to a decrease in conversion [27].

#### 3.4.3. Effect of Temperature

**Figure 3:**
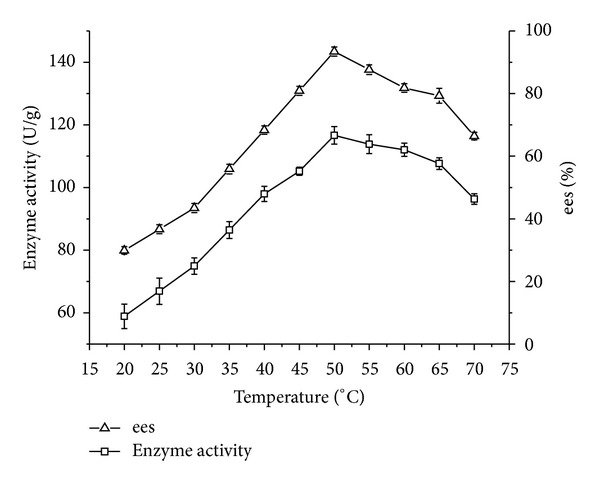
Effect of temperature on enzyme activity/ees of CRL7. Reaction condition: 0.1 g CRL7 was added to 5 mL isooctane containing 0.1 mmol ibuprofen, 1 mmol isooctanol. The reactions were performed at different temperatures, 200 rpm for 24 h. The data were measured in triplicate and vertical bars represent standard deviation.

For analysis of temperature influence, reactions were carried out within the range from 20 to 70°C. As shown in Figure 3, when temperature was below 50°C, enzyme activity and ees showed an increasing tendency with the increase of temperature. This increase can be explained by temperature dependency of the reaction rate. When temperature was beyond 50°C, the further increase of temperature would result in a decrease in both enzyme activity and ees, indicating that too much higher temperature had negative effect on enzyme activity and ees.

#### 3.4.4. Effect of Reaction Time

**Figure 4:**
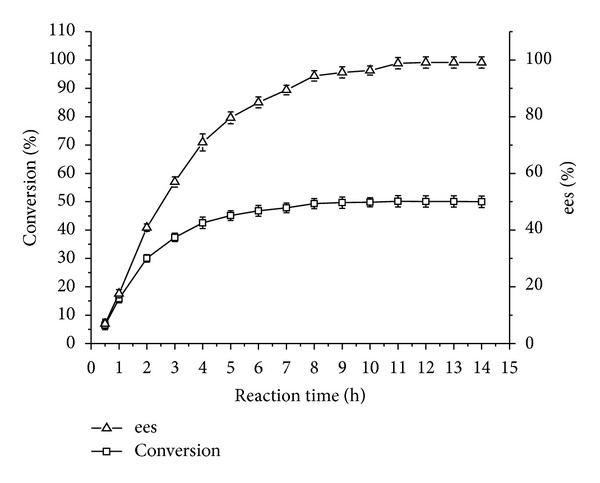
Effect of reaction time on conversion/ees of CRL7. Reaction condition: 0.1 g CRL7 was added to 5 mL isooctane containing 0.1 mmol ibuprofen, 1 mmol isooctanol. The reactions were performed at 50°C, 200 rpm for different reaction times. The data were measured in triplicate and vertical bars represent standard deviation.

As shown in Figure 4, when reaction time was more than 20 h, the conversion and ees were close to 50% and 100%, respectively, and the corresponding *E* value was more than 200. It indicated that all of (*S*)-ibuprofen had nearly been converted into (*S*)-ibuprofen isooctyl ester, while (*R*)-ibuprofen remained unchanged in the reaction mixture, which also further proved that CRL7 had a good preference for (*S*)-ibuprofen. Moreover, it had been reported that the unreacted ibuprofen and the corresponding ester could be quantitatively separated by bulb-to-bulb distillation because of the molecular weight difference between them [28].

## 4. Conclusion

In this study, according to the methods of substrate engineering and medium engineering, it could be stated that alcohols and solvents had great effect on the enantioselective performance of CRL7. The effects of carbon chain length of alcohols were larger than solvents on enzyme activity and enantioselectivity. 1-Isooctanol and isooctane were recommended for the best substrate and best reaction medium, respectively, because of the highest enantioselectivity. The investigation of reaction parameters (such as molar ratio, water content, temperature, and reaction time) showed that CRL7 had a good preference for (*S*)-ibuprofen and a great prospect in industrial application.

## Figures and Tables

**Table 1:** Effect of alcohols on the enzymatic esterification of ibuprofen*.

	Acyl donor	Enzyme activity (U/g)	ees (%)
1	Methanol	41.05 ± 0.65	24.11 ± 0.06
2	Ethanol	44.16 ± 1.20	12.12 ± 0.03
3	1-Propanol	33.05 ± 1.57	16.22 ± 0.08
4	1-Butanol	84.50 ± 1.12	53.42 ± 0.06
5	1-Pentanol	95.87 ± 0.75	66.41 ± 0.11
6	1-Hexanol	88.11 ± 0.93	54.50 ± 0.12
7	1-Heptanol	91.29 ± 0.58	61.01 ± 0.12
8	1-Octanol	91.43 ± 0.56	69.42 ± 0.07
9	1-Nonanol	103.9 ± 3.91	73.91 ± 0.05
10	1-Decanol	106.8 ± 2.56	83.51 ± 0.03
11	Isobutanol	93.61 ± 2.56	74.91 ± 0.05
12	Isoamylol	108.95 ± 2.56	87.51 ± 0.06
13	Isooctanol	115.46 ± 3.12	95.72 ± 0.04
14	*tert*-Butanol	ND	ND
15	*tert*-Amyl alcohol	ND	ND
16	1,2-Ethanediol	ND	ND

*The reactions were performed at 50°C, 200 rpm for 12 h. 0.1 g CRL7 was added to 5 mL isooctane containing 0.1 mmol ibuprofen, 1.0 mmol alcohol (from C1 to C10). The data were measured in triplicate and expressed in mean ± standard deviation (SD); ND indicates not determined.

**Table 2:** Effect of solvents on the enzyme activity and ees via esterification of ibuprofen*.

	Solvent	log⁡*P*	Enzyme activity (U/g)	ees (%)
1	*n*-Undecane	6.1	82.67 ± 1.56	49.89 ± 0.02
2	*n*-Decane	5.6	74.55 ± 1.51	41.57 ± 0.02
3	*n*-Nonane	5.1	64.27 ± 4.21	35.39 ± 0.01
4	Isooctane	4.7	118.02 ± 5.65	92.01 ± 0.01
5	*n*-Octane	4.5	79.70 ± 3.13	43.75 ± 0.02
6	*n*-Heptane	4.0	79.72 ± 4.15	45.51 ± 0.01
7	*n*-Hexane	3.5	81.24 ± 6.41	42.38 ± 0.02
8	Cyclohexane	3.2	79.01 ± 5.65	43.83 ± 0.01
9	Xylene	2.5	23.84 ± 5.35	8.54 ± 0.04
10	Acetonitrile	−0.33	ND	ND
11	BmimTF2N		60.68 ± 4.64	33.44 ± 0.01
12	BmimPF6		84.95 ± 3.23	53.96 ± 0.02
13	EmimPF6		96.42 ± 5.11	65.84 ± 0.01

*The reactions were performed at 50°C, 200 rpm for 12 h. 0.1 g CRL7 was added to 5 mL solvent (from log⁡*P* = 6.1 to log⁡*P* = −0.33) containing 0.01 mmol ibuprofen, 1.0 mmol isooctanol. The data were measured in triplicate and expressed in mean ± standard deviation (SD); ND indicates not determined.

**Table 3:** Quantitative estimation of the secondary structure elements of the treated CRL7 calculated by FT-IR spectroscopy measurement*.

Solvent	*α*-Helix (%)	*β*-Sheet (%)	*β*-Turn (%)	Random coil (%)
Control	43.46 ± 0.02	26.91 ± 0.02	11.83 ± 0.03	17.79 ± 0.04
*n*-Undecane	33.39 ± 0.02	27.10 ± 0.09	21.57 ± 0.02	17.94 ± 0.02
*n*-Decane	21.38 ± 0.21	46.04 ± 0.11	11.93 ± 0.06	20.65 ± 0.15
*n*-Nonane	21.03 ± 0.03	46.43 ± 0.03	12.76 ± 0.13	19.78 ± 0.07
Isooctane	39.03 ± 0.12	37.61 ± 0.04	13.53 ± 0.06	18.72 ± 0.05
*n*-Octane	23.62 ± 0.06	40.95 ± 0.16	13.75 ± 0.12	21.68 ± 0.08
*n*-Heptane	22.23 ± 0.13	44.55 ± 0.15	13.04 ± 0.14	20.18 ± 0.06
*n*-Hexane	38.53 ± 0.09	27.83 ± 0.13	13.81 ± 0.09	19.83 ± 0.10
Cyclohexane	21.17 ± 0.12	45.74 ± 0.12	12.82 ± 0.04	20.26 ± 0.10
Xylene	37.43 ± 0.05	22.22 ± 0.12	14.51 ± 0.10	25.83 ± 0.15
Acetonitrile	40.88 ± 0.12	24.94 ± 0.04	14.57 ± 0.13	19.59 ± 0.02
BmimTF2N	24.27 ± 0.05	25.62 ± 0.10	21.58 ± 0.11	28.52 ± 0.11
BmimPF6	24.90 ± 0.04	14.39 ± 0.09	19.43 ± 0.12	41.28 ± 0.14
EmimPF6	32.33 ± 0.10	23.09 ± 0.08	16.81 ± 0.13	27.77 ± 0.12

*The lipase without treatment of organic solvent and ionic liquids was set as control.

The data were measured in triplicate and expressed in mean ± standard deviation (SD).
